# Underestimated Peripheral Effects Following Pharmacological and Conditional Genetic Microglial Depletion

**DOI:** 10.3390/ijms21228603

**Published:** 2020-11-15

**Authors:** Jinming Han, Yueshan Fan, Kai Zhou, Keying Zhu, Klas Blomgren, Harald Lund, Xing-Mei Zhang, Robert A. Harris

**Affiliations:** 1 Applied Immunology and Immunotherapy, Department of Clinical Neuroscience, Karolinska Institutet, Center for Molecular Medicine, Karolinska University Hospital, S-171 76 Stockholm, Sweden; fanyueshan@hotmail.com (Y.F.); keying.zhu@ki.se (K.Z.); harald.lund@ki.se (H.L.); xingmei.zhang@ki.se (X.-M.Z.); 2 Department of Women’s and Children’s Health, Karolinska Institutet, Karolinska University Hospital, S-171 76 Stockholm, Sweden; kai.zhou@ki.se (K.Z.); klas.blomgren@ki.se (K.B.); 3 Pediatric Oncology, Karolinska University Hospital, S-171 76 Stockholm, Sweden; 4 Department of Physiology and Pharmacology, Karolinska Institutet, Center for Molecular Medicine, Karolinska University Hospital, S-171 76 Stockholm, Sweden

**Keywords:** monocytes, tissue macrophages, colony-stimulating factor 1 receptor inhibitor

## Abstract

Microglia, predominant parenchymal resident macrophages in the central nervous system (CNS), are crucial players in neurodevelopment and CNS homeostasis. In disease conditions, pro-inflammatory microglia predominate over their regulatory counterparts, and are thus a potential immunotherapeutic target. It has been well documented that microglia can be effectively depleted using both conditional genetic *Cx3cr1*^Cre^-diphtheria toxin receptor (DTR)/diphtheria toxin subunit A (DTA) animal models and pharmacological colony-stimulating factor 1 receptor (CSF1R) inhibitors. Recent advances using these approaches have expanded our knowledge of the multitude of tasks conducted by microglia in both homeostasis and diseases. Importantly, experimental microglial depletion has been proven to exert neuroprotective effects in an increasing number of disease models, mostly explained by reduced neuroinflammation. However, the comprehensive effects of additional targets such as circulating monocytes and peripheral tissue macrophages during microglial depletion periods have not been investigated widely, and for those studies addressing the issue the conclusions are mixed. In this study, we demonstrate that experimental microglial depletion using both *Cx3cr1*^CreER/+^*Rosa26*^DTA/+^ mice and different doses of CSF1R inhibitor PLX3397 exert crucial influences on circulating monocytes and peripheral tissue macrophages. Our results suggest that effects on peripheral immunity should be considered both in interpretation of microglial depletion studies, and especially in the potential translation of microglial depletion and replacement therapies.

## 1. Introduction

Microglia are resident macrophages in the central nervous system (CNS), acting as key players in immune surveillance, neural circuits and synapse formation [1]. Growing evidence points toward a critical role of reactive microglia in disease conditions through their production of inflammatory mediators [2]. Conditional genetic and pharmacological tools have been widely used in order to ablate microglia in research settings, as we have previously reviewed [3,4,5]. These studies have expanded our understanding of microglial biology in both homeostasis and diseases.

Microglia are characterized by high expression of the chemokine receptor CX3CR1. *Cx3cr1* is widely used to genetically label microglia and the Cre/loxP system is an applied technology for site-specific genetic manipulation in preclinical animal models [6]. Specifically, *Cx3cr1*^Cre^-diphtheria toxin receptor (DTR) mouse, with a tamoxifen-inducible Cre-recombinase expressed under control of the *Cx3cr1* promoter, was bred in order to deplete microglia after the activation of tamoxifen and intraperitoneal administration of diphtheria toxin [7]. This conditional genetic approach can deplete approximately 80% of microglia in the mouse brain [7]. In addition, treatment of *Cx3cr1*^CreER/+^*Csf1r*^Flox/Flox^ mice with a 12-week tamoxifen diet resulted in partial depletion of microglia [8]. We have previously reported that microglia are efficiently eliminated by administration of tamoxifen alone in *Cx3cr1*^CreER/+^*Rosa26*^diphtheria toxin subunit A (DTA)/+^ mice, leading to more than 90% depletion of microglia [9,10]. However, it is important to note that other peripheral immune cells including activated T cells and NK cells also express CX3CR1 [11].

The survival, maintenance and proliferation of microglia are profoundly dependent on the colony-stimulating factor 1 receptor (CSF1R). CSF1R is a receptor tyrosine kinase which is mainly expressed on microglia within the CNS. The pharmacological targeting of CSF1R using compounds including PLX3397 [12], PLX5622 [13], BLZ945 [14], Ki20227 [15] and GW2850 [16] has been widely used in order to ablate microglia in both preclinical and clinical settings [17]. CSF1R inhibitors at different doses (290 mg/kg or 1200 mg/kg) can be added into diets and continuous dietary administration of these compounds to mice leads to effective microglial depletion [18]. Specifically, PLX3397 at a dose of 290 mg/kg for 14 consecutive days can deplete more than 80% of microglia in the spinal cord [18]. PLX5622, a more specific CSF1R inhibitor that does not inhibit c-Kit, at a dose of 1200 mg/kg for 7 days can eliminate over 85% of microglia in the brain [19]. Again, systematic administration of these CSF1R inhibitors may exert potential effects on peripheral immune cells and cause side-effects. In support of this notion, CSF1R-related leukoencephalopathy, a disease mainly caused by CSF1R gene mutations, can lead to a marked decreased frequency of circulating non-classical monocytes during disease progression [20].

Previous studies using currently available depletion approaches have mainly assessed effects on microglia in the CNS, and a few studies have reported that CSF1R inhibitors did not significantly alter leukocyte populations in the circulation [18,21]. For example, it has been recorded that PLX3397 at a concentration of 290 mg/kg does not affect the numbers and proportions of macrophages and T cells in both the spleen and draining lymph nodes [22]. These results implicate that CSF1R inhibitors specifically target microglia in the CNS, and reference to influences on peripheral immunity (without detailed testing) have been made [23]. However, emerging data has inferred the opposite conclusion that the gene expression *csf1r* of liver tissue macrophages can be reduced using both *Cx3cr1*-DTR rats and PLX5622 treatment [24]. PLX5622 diet at a concentration of 1200 mg/kg for 21 days, frequently used in research settings, could reduce the numbers of macrophages and monocytes in the periphery [25]. Furthermore, treatment of *Cx3cr1*^CreER/+^*Csf1r*^Flox/Flox^ mice with tamoxifen caused an obvious deficiency of intestinal macrophages [8]. It has also been recently proposed that PLX5622 is not microglia specific and can also exert significant influences on peritoneum, lung, and liver tissue macrophages, with the splenic populations being less affected [26]. PLX5622 treatment also exerts long-term effects on hematopoietic progenitor cells and hematopoietic stem cells, affecting hematopoiesis [26].

Given this controversy in the research field, the objective of the present study was to confirm if experimental elimination of microglia using additional methods such as conditional genetic *Cx3cr1*^CreER/+^*Rosa26*^DTA/+^ mice and CSF1R inhibition (PLX3397) exerts effects on peripheral immunity with a focus on the spleen.

## 2. Results

### 2.1. Microglia Are Effectively Depleted 21 Days after PLX3397 Treatment

**Figure 1:**
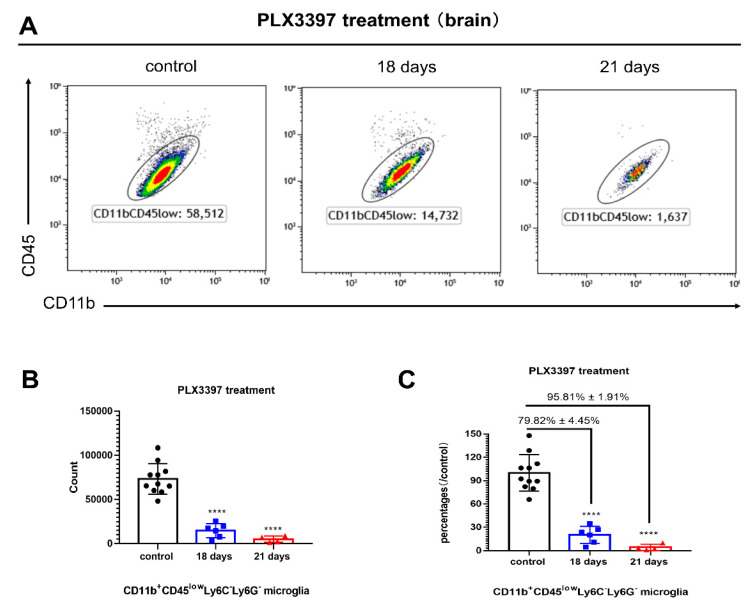
Microglia are effectively depleted 21 days following PLX3397 treatment. (**A**) Representative flow cytometry plots of CD11b^+^CD45^low^Ly6C^−^Ly6G^−^ microglia of the hemi-brains in *C57BL/6NTac* mice following consecutive PLX3397 diet (18 and 21 days) at a dose of 290 mg/kg. Control mice were treated with normal diet. (**B**) Total CD11b^+^CD45^low^Ly6C^−^Ly6G^−^ microglial counts (± SEM) of the hemi-brains during microglial depletion periods (control, black bars; day 18, blue bars; day 21, red bars). (**C**) Percentages of CD11b^+^CD45^low^Ly6C^−^Ly6G^−^ microglia (/control, ± SEM) during microglial depletion periods (control, black bars; day 18, blue bars; day 21, red bars, *n* = 11, 6, 4, respectively). Statistical significance is indicated as **** *p* < 0.0001.

We have previously reported that CD11b^+^CD45^low^Ly6C^−^Ly6G^−^ microglia can be effectively depleted (95%) 7 days after the administration of tamoxifen in *Cx3cr1*^CreER/+^*Rosa26*^DTA/+^ mice [9,10]. In the current study, PLX3397 standard diet (290 mg/kg) was administered for up to 21 consecutive days in *C57BL/6NTac* mice, with control mice receiving a normal diet. Mice were terminated 18 or 21 days following PLX3397 treatment. No obvious side-effects were observed with PLX3397, except for whitening of fur color as previously reported [21]. Flow cytometric analyses of brain tissues were performed at each time point. Our results demonstrated that CD11b^+^CD45^low^Ly6C^−^Ly6G^−^ microglia can be effectively depleted 18 days (79.82% ± 4.45%) and 21 days (95.81% ± 1.91%) following PLX3397 treatment at a concentration of 290 mg/kg (Figure 1A–C, **** *p* < 0.0001).

### 2.2. Splenic Red Pulp Macrophages Are Significantly Decreased Following Both Conditional Genetic and Pharmacological Microglial Depletion

**Figure 2:**
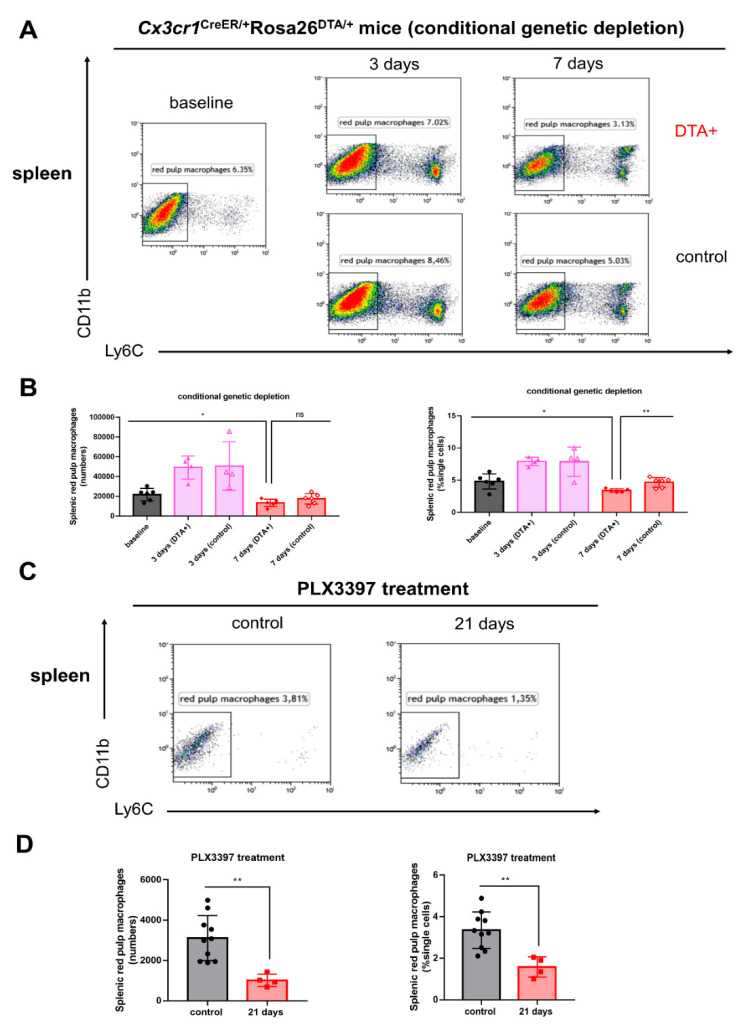
Splenic red pulp macrophages are reduced following both conditional genetic and pharmacological microglial depletion. (**A**) Representative flow cytometry plots of splenic red pulp macrophages in *Cx3cr1*^CreER/+^*Rosa26*^DTA/+^ and *Cx3cr1*^CreER/+^ mice at days 3 and 7 post tamoxifen injections. Red pulp macrophages were analyzed under the F4/80^hi^ gate. (**B**) The numbers and percentages of splenic red pulp macrophages (± SEM) during conditional genetic microglial depletion periods (baseline, black bars; day 3, pink bars; day 7, red bars, *n* = 6, 4, 4, 6, 6, respectively). (**C**) Representative flow cytometry plots of splenic red pulp macrophages in *C57BL/6NTac* mice following 290 mg/kg PLX3397 diet (21 days). Control mice were treated with normal diet. (**D**) The numbers and percentages of splenic red pulp macrophages (± SEM) during pharmacological microglial depletion periods (control, black bars; day 21, red bars, *n* = 10, 4, respectively). A total of 400,000 cells from the spleen were run for flow cytometry in this experiment. Statistical significance is indicated as * *p* < 0.05 and ** *p* < 0.01 (ns: not significant).

We next addressed whether splenic red pulp macrophages were influenced after conditional genetic and pharmacological microglial depletion. *Cx3cr1*^CreER/+^*Rosa26*^DTA/+^ and *Cx3cr1*^CreER/+^ mice were treated with tamoxifen in order to induce the Cre recombinase and mice were terminated 3 or 7 days later. The splenic flow cytometry gating strategy is depicted in Supplementary Figure S1. We determined that in *Cx3cr1*^CreER/+^*Rosa26*^DTA/+^ mice the numbers and proportions of F4/80^hi^Ly6C^−^splenic red pulp macrophages were significantly decreased 7 days after tamoxifen treatment, the most significant time point of depletion (Figure 2A,B, * *p* < 0.05, ** *p* < 0.01). Similar findings with reduced numbers and percentages of F4/80^hi^Ly6C^−^ splenic red pulp macrophages were also noted using 290 mg/kg PLX3397 diet pharmacological-induced microglial depletion. Our results discerned that the numbers and percentages of F4/80^hi^Ly6C^−^ red pulp macrophages in the spleen were significantly fewer 21 days following PLX3397 treatment, the most significant time point of pharmacological microglial depletion, than control group (Figure 2C,D ** *p* < 0.01).

### 2.3. Splenic Ly6C^hi^ Monocytes Are Significantly Increased Following Conditional Genetic Microglial Depletion, but Not Pharmacological Depletion

**Figure 3:**
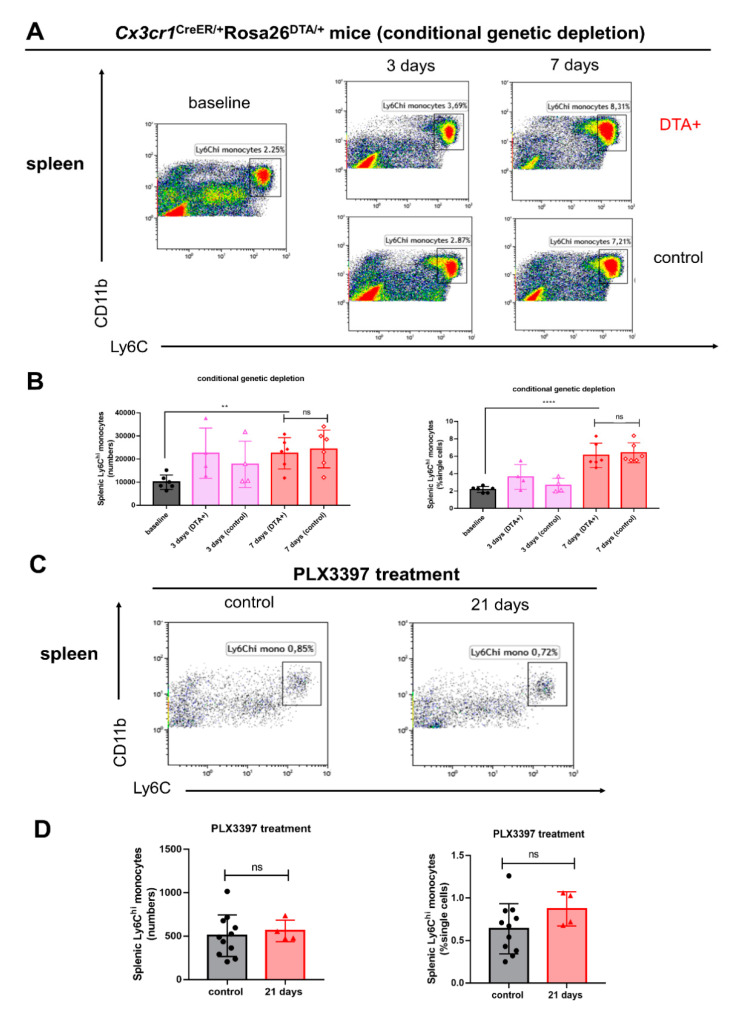
Splenic Ly6C^hi^ monocytes are increased following conditional genetic microglial depletion, but not after pharmacological depletion. (**A**) Representative flow cytometry plots of CD11b^+^Ly6G^−^Ly6C^hi^ splenic monocytes in *Cx3cr1*^CreER/+^*Rosa26*^DTA/+^ and *Cx3cr1*^CreER/+^ mice at days 3 and 7 post tamoxifen injections. (**B**) The numbers and percentages of splenic Ly6C^hi^ monocytes (± SEM) in the spleen during conditional genetic microglial depletion periods (baseline, black bars; day 3, pink bars; day 7, red bars, *n* = 6, 4, 4, 6, 6, respectively). (**C**) Representative flow cytometry plots of splenic Ly6C^hi^ monocytes in *C57BL/6NTac* mice following PLX3397 diet (21 days) at a dose of 290 mg/kg. Control mice were treated with normal diet. (**D**) The numbers and percentages of splenic Ly6C^hi^ monocytes (± SEM) during pharmacological microglial depletion periods (control, black bars; day 21, red bars, *n* = 11, 4, respectively). Statistical significance is indicated as ** *p* < 0.01 and **** *p* < 0.0001 (ns: not significant).

We next addressed whether splenic Ly6C^hi^ monocytes could also be influenced following both genetic and pharmacological microglial depletion. Unlike reduced numbers of splenic red pulp macrophages, we demonstrated that during conditional genetic microglial depletion periods the numbers and percentages of splenic Ly6C^hi^ monocytes were significantly increased 7 days following tamoxifen treatment (Figure 3A,B **** *p* < 0.0001; ** *p* < 0.01). The numbers and percentages of splenic Ly6C^hi^ monocytes were not significantly different between *Cx3cr1*^CreER/+^*Rosa26*^DTA/+^ mice and *Cx3cr1*^CreER/+^ mice (Figure 3B). Furthermore, 290 mg/kg PLX3397-mediated microglial depletion did not significantly alter the numbers and percentages of splenic Ly6C^hi^ monocytes (Figure 3C,D).

### 2.4. Splenic CD4^+^ T Cells and NK Cells Are Affected Following Conditional Genetic and Pharmacological Microglial Depletion

**Figure 4:**
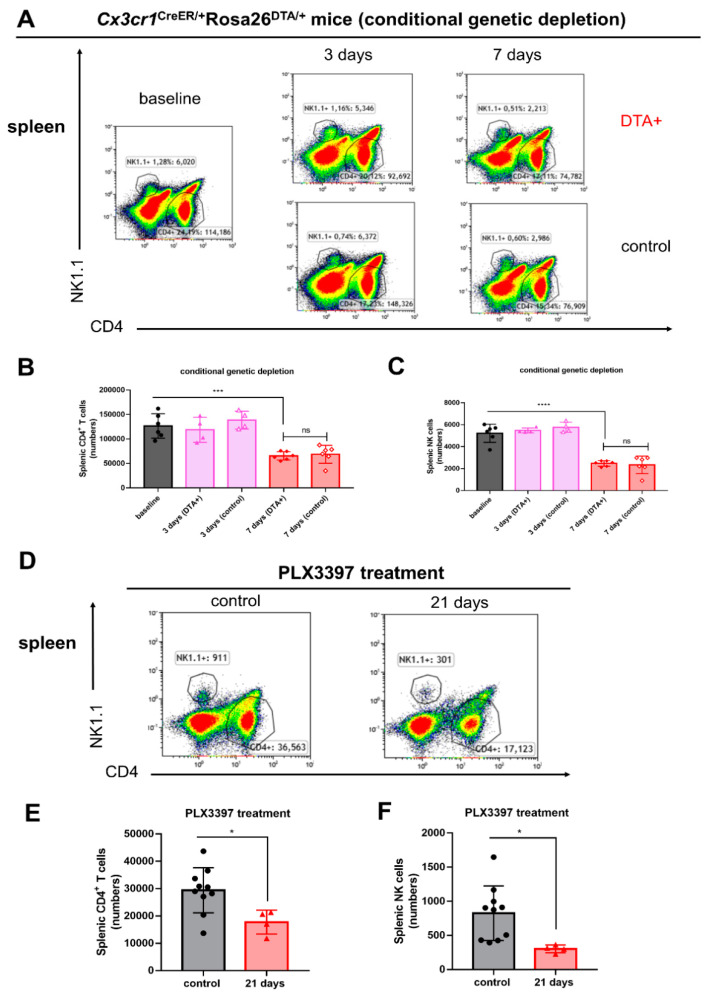
Splenic CD4^+^ T cells and NK cells are affected following conditional genetic and pharmacological microglial depletion. (**A**) Representative flow cytometry plots of splenic CD4^+^ T cells and NK1.1^+^ cells in *Cx3cr1*^CreER/+^*Rosa26*^DTA/+^ and *Cx3cr1*^CreER/+^ mice 3 and 7 days after tamoxifen injections. (**B**,**C**) Quantitative analysis of splenic CD4^+^ T cells and NK1.1^+^ cells (± SEM) during conditional genetic microglial depletion periods (baseline, black bars; day 3, pink bars; day 7, red bars, *n* = 6, 4, 4, 6, 6, respectively). (**D**) Representative flow cytometry plots of splenic CD4^+^ T cells and NK1.1^+^ cells in *C57BL/6NTac* mice following PLX3397 diet (21 days) at a dose of 290 mg/kg. Control mice were treated with normal diet. (**E**,**F**) Quantitative analysis of splenic CD4^+^ T cells and NK1.1^+^ cells (± SEM) during pharmacological microglial depletion periods (control, black bars; day 21, red bars, *n* = 10, 4, respectively). Statistical significance is indicated as * *p* < 0.05, *** *p* < 0.001 and **** *p* < 0.0001 (ns: not significant).

Our results demonstrated that during both conditional genetic and pharmacological microglial depletion periods the numbers of splenic CD4^+^ T cells were significantly decreased after 7 days (Figure 4A,B *** *p* < 0.001) and 21 days (Figure 4D,E * *p* < 0.05), respectively. Furthermore, the numbers and percentages of splenic NK cells were significantly reduced following both conditional genetic and pharmacological microglial depletions (Figure 4C,F **** *p* < 0.0001, * *p* < 0.05).

### 2.5. Reduced Numbers of Monocytes in the Circulation Following Conditional Genetic and Pharmacological Microglial Depletion

**Figure 5:**
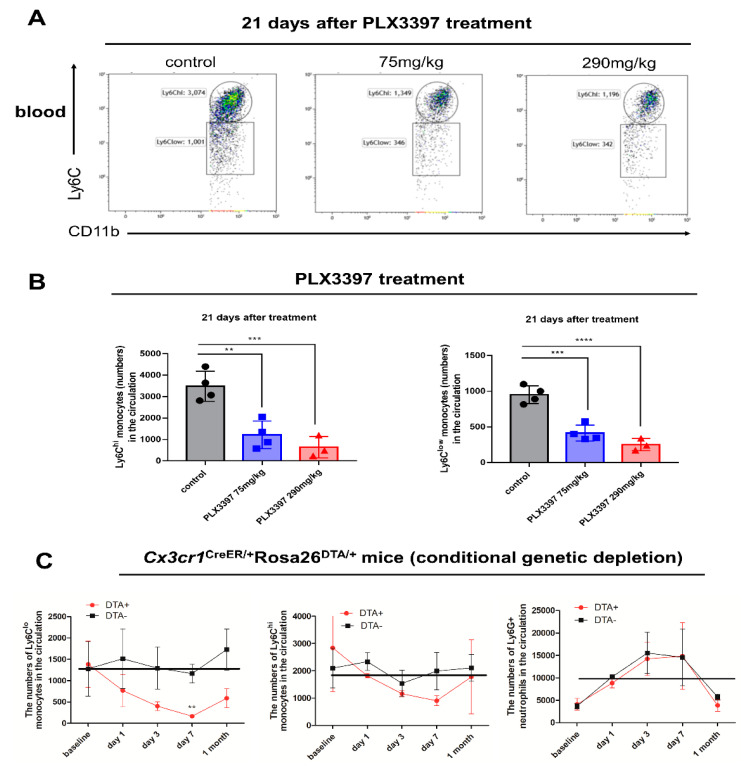
The numbers of circulating monocytes are reduced following both conditional genetic and pharmacological microglial depletion. (**A**) Representative flow cytometry plots of Ly6C^hi^ and Ly6C^low^ monocytes in the circulation following 21 consecutive days of PLX3397 diet treatment at different doses of 75 mg/kg and 290 mg/kg, respectively. Control mice were treated with normal diet. (**B**) The numbers of circulating Ly6C^hi^ and Ly6C^low^ monocytes (± SEM) during pharmacological microglial depletion periods (control, black bars; 75 mg/kg PLX3397 treatment, blue bars; 290 mg/kg PLX3397 treatment, red bars, *n* = 4, 4, 3, respectively). (**C**) Kinetic changes of circulating Ly6C^hi^ monocytes, Ly6C^low^ monocytes and Ly6G^+^ neutrophils in *Cx3cr1*^CreER/+^*Rosa26*^DTA/+^ mice treated with tamoxifen (baseline, *n* = 6; *Cx3cr1*^CreER/+^ mice, black bars; *n* = 4, 4, 4, 5, respectively; *Cx3cr1*^CreER/+^*Rosa26*^DTA/+^ mice, red bars; *n* = 3, 4, 4, 3, respectively). Statistical significance is indicated as ** *p* < 0.01, *** *p* < 0.001 and **** *p* < 0.0001 (ns: not significant).

We explored the dynamic changes of circulating monocytes following conditional genetic and pharmacological microglial depletions. The blood gating strategy is depicted in Supplementary Figure S2. Our results indicated that the numbers of both circulating classical Ly6C^hi^ monocytes and non-classical Ly6C^low^ monocytes were significantly decreased even when using the PLX3397 research diet at a lower concentration of 75 mg/kg (Figure 5A,B ** *p* < 0.01, *** *p* < 0.001 and **** *p* < 0.0001). For conditional genetic microglial depletion, the numbers of circulating Ly6G^+^ neutrophils were gradually increased in both groups as a result of tamoxifen injections (Figure 5C). The numbers of circulating Ly6G^+^ neutrophils reached the highest levels after 7 days, but returned to the baseline level one month later (Figure 5C). We also observed that numbers of circulating classical Ly6C^hi^ monocytes and non-classical Ly6C^low^ monocytes in the circulation were gradually decreased 7 days after tamoxifen injections (Figure 5C ** *p* < 0.01). Interestingly, while classical Ly6C^hi^ monocyte numbers returned to the baseline level one month later, non-classical Ly6C^low^ monocytes did not (Figure 5C). No significant differences in numbers of circulating classical Ly6C^hi^ monocytes and non-classical Ly6C^low^ monocytes were noted in *Cx3cr1*^CreER/+^ control mice during microglial depletion periods (Figure 5C).

### 2.6. Dose-Dependent Peripheral Effects Following Pharmacological Microglial Depletion

**Figure 6:**
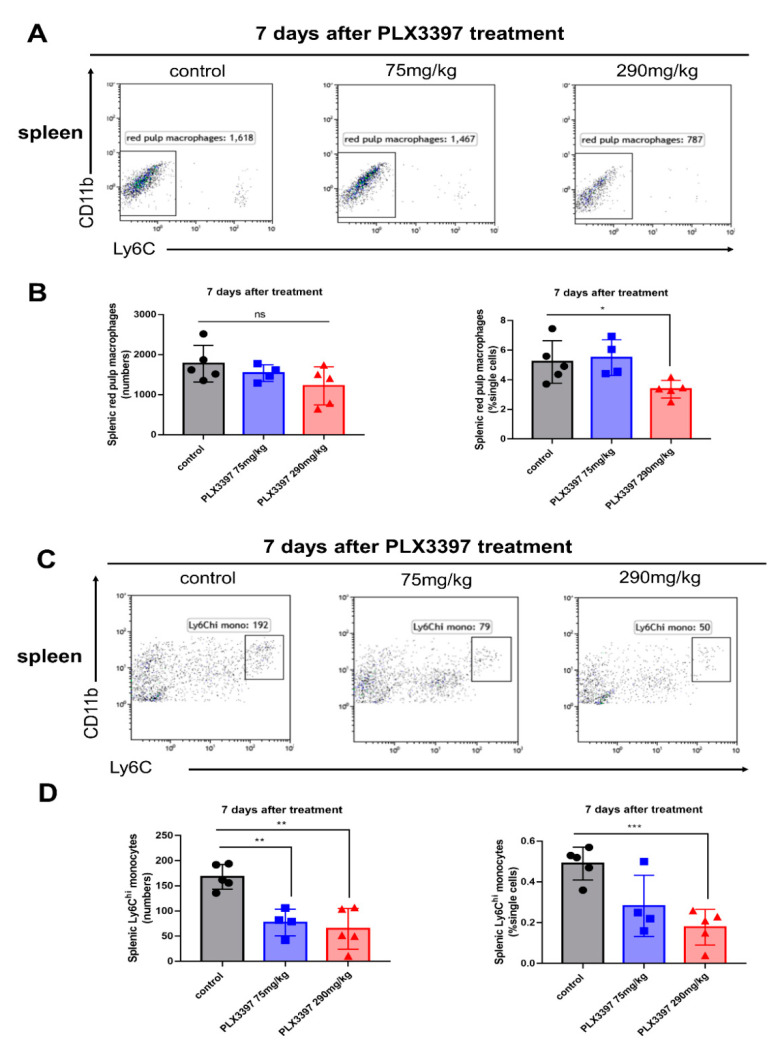
Dose-dependent peripheral effects following pharmacological microglial depletion. (**A**) Representative flow cytometry plots of splenic red pulp macrophages following 7 consecutive days of PLX3397 diet treatment with different doses of 75 mg/kg and 290 mg/kg, respectively. Control mice were treated with normal diet. (**B**) The numbers and percentages of red pulp macrophages (± SEM) in the spleen following PLX3397 diet with different doses (control, black bars; 75 mg/kg PLX3397 diet, blue bars; 290 mg/kg PLX3397 diet, red bars, *n* = 5, 4, 5, respectively). (**C**) Representative flow cytometry plots of splenic Ly6C^hi^ monocytes following 7 consecutive days of PLX3397 diet treatment with different doses. Control mice were treated with normal diet. (**D**) The numbers and percentages of splenic Ly6C^hi^ monocytes (± SEM) following PLX3397 with different doses (control, black bars; 75 mg/kg PLX3397 diet, blue bars; 290 mg/kg PLX3397 diet, red bars, *n* = 5, 4, 5, respectively). Statistical significance is indicated as * *p* < 0.05, ** *p* < 0.01 and *** *p* < 0.001.

We demonstrated that both splenic red pulp macrophages and circulating monocytes could be affected during microglial depletion. Given that different concentrations of pharmacological inhibitors have previously been used in different previous studies, we next explored whether these peripheral effects were concentration-dependent and occurred at an earlier time point. *C57BL/6NTac* mice were thus fed with two distinct doses of PLX3397 diet (290 mg/kg and 75 mg/kg). The lower dose of PLX3397 treatment failed to effectively deplete microglia (Supplementary Figure S3). Consistent with our previous findings, we observed that the percentages splenic red pulp macrophages were significantly reduced 7 days after 290 mg/kg PLX3397 treatment, but not in the 75 mg/kg low dose group (Figure 6A,B * *p* < 0.05). Clearly, the numbers and proportions of splenic monocytes were significantly reduced 7 days after PLX3397 treatment with a dose of 290 mg/kg (Figure 6C,D ** *p* < 0.01; *** *p* < 0.001). Additionally, the numbers of splenic monocytes were also significantly decreased with the lower dose PLX3397 diet (Figure 6D ** *p* < 0.01).

### 2.7. Intracisternal Injection of PLX3397 Does Not Exert Significant Influence on Splenic Myeloid Cells

**Figure 7:**
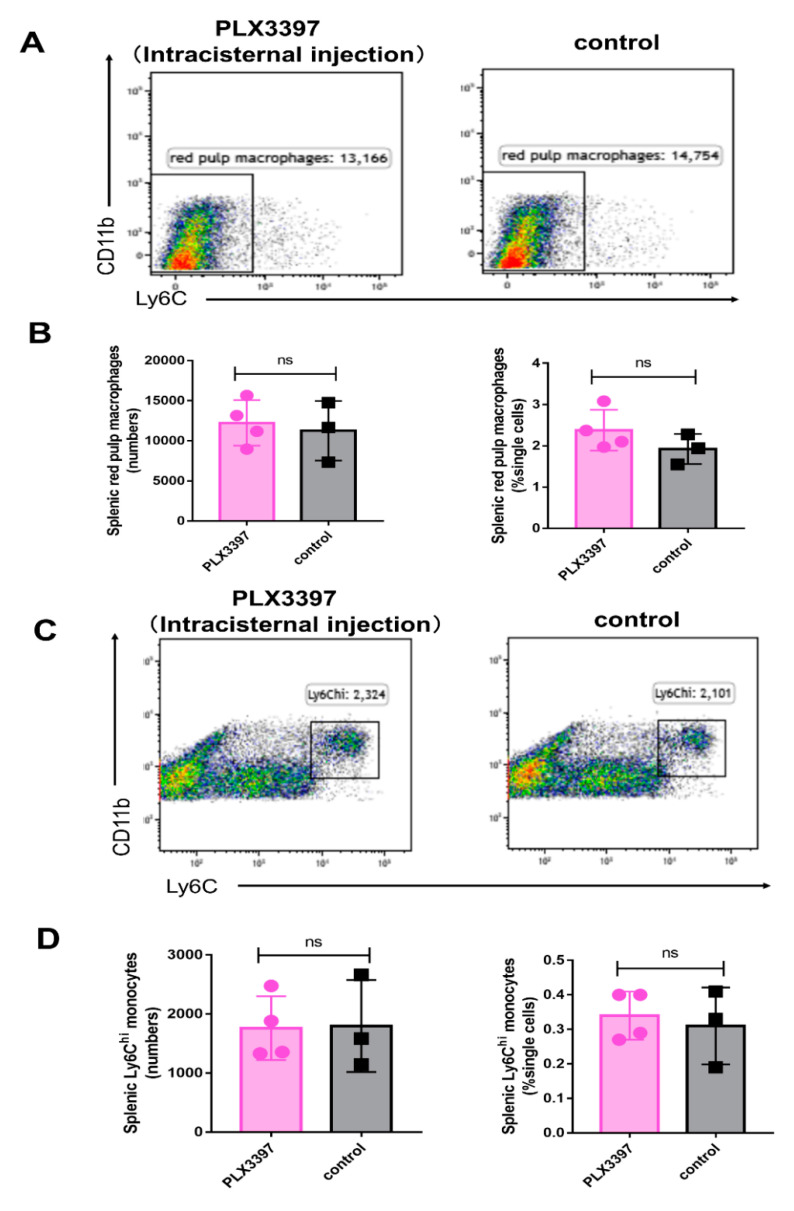
Intracisternal injection of PLX3397 does not exert a significant effect on splenic myeloid cells. (**A**,**B**) The number and percentage of red pulp macrophages (± SEM) in the spleen following intracisternal injection of PLX3397 (PLX3397 treatment group, pink bars; control, black bars, *n* = 4, 3, respectively). (**C**,**D**) The number and percentage of splenic Ly6C^hi^ monocytes (± SEM) following intracisternal PLX3397 treatment (PLX3397 treatment group, pink bars; control, black bars, *n* = 4, 3, respectively). ns: not significant.

To avoid unnecessary peripheral effects during microglial depletion we next assessed if intracisternal injection of PLX3397 (10 μL per injection for two consecutive days) had any effects on splenic myeloid cells. However, this method caused only a partial depletion of microglia (not shown). Unlike systemic administration of the PLX3397 diet, the numbers and percentages of splenic red pulp macrophages (Figure 7A,B) and monocytes (Figure 7C,D) did not alter significantly following intracisternal injections of PLX3397.

## 3. Discussion

In this study, we demonstrated that both conditional genetic and pharmacological microglial depletion approaches have significant effects on circulating monocytes and peripheral tissue macrophages, and that these effects can be abrogated by direct delivery of pharmacological inhibitors directly into the CNS. These findings should be taken into due consideration in the interpretation of microglial depletion results and in the planning for clinical translation of microglial replacement therapy.

Unlike other tissue macrophages derived from the bone marrow and fetal liver, microglia arise exclusively from the yolk sac during early neurodevelopment [27], exhibiting a distinct transcriptomic profile (including P2ry12 and Tmem119) [28]. Recent sophisticated analysis of protein-coding genes across different species convincingly demonstrated that a variety of microglial signature genes are also highly enriched in the selective peripheral organs, such as in the circulation and lymphoid tissues [29].

It is documented that classical monocytes have a low expression of CX3CR1, while non-classical monocytes exhibit high expression. In support of this, our results demonstrated that numbers of circulating classical Ly6C^hi^ monocytes and non-classical Ly6C^low^ monocytes significantly decreased in *Cx3cr1*^CreER/+^*Rosa26*^DTA/+^ mice following tamoxifen treatment, with non-classical Ly6C^low^ monocytes being most affected. Consistent with this idea, systemic administration of a high-affinity inhibitor of CX3CR1 (AZD8797) can effectively block infiltrating CX3CR1^+^ leukocytes, but not CX3CR1-expressing microglia in the CNS, subsequently relieving clinical symptoms and inhibiting the progression of experimental autoimmune encephalomyelitis, an animal model of multiple sclerosis [30]. Furthermore, the spleen contains a reservoir of monocytes which can be released upon tissue injury. One previous study reported that 1 day after the administration of diphtheria toxin in *Cx3cr1*-DTR mice, the numbers of activated macrophages in the cochlea and spleen following systemic lipopolysaccharide injections were significantly reduced, suggesting that peripheral CX3CR1-expressing macrophages were affected using this conditional genetic tool [11]. In our current study, peripheral effects were measured within one week after tamoxifen treatment, with splenic Ly6C^hi^ monocytes being increased in both *Cx3cr1*^CreER/+^*Rosa26*^DTA/^^+^ and *Cx3cr1*^CreER/+^ mice. At the same time, circulating Ly6G^+^ neutrophils reached the highest levels 7 days after tamoxifen injections. In this case, peripheral effects caused by tamoxifen alone cannot be ruled out in our study. Our results also indicated that both the numbers and percentages of splenic red pulp macrophages were significantly decreased in *Cx3cr1*^CreER/+^*Rosa26*^DTA/+^ mice following tamoxifen treatment, indicating that tissue resident macrophages can be affected following conditional genetic microglial depletion. Further studies using conditional genetic microglial depletion including *Cx3cr1*^CreER/+^*Rosa26*^DTA/+^ and *Cx3cr1*-DTR are needed to verify our findings in other peripheral organs.

The survival, maintenance and proliferation of microglia is critically dependent on close interplay between CSF-1 and its receptor CSF1R. This receptor is a tyrosine kinase, and despite mainly being expressed on microglia in the CNS, it can also be expressed on other tissue macrophages and peripheral monocytes. Some previous studies concluded that systemic administration of CSF1R inhibitors exerted limited influence on other immune cells in the periphery [23], with dietary PLX3397 at a dose of 290 mg/kg not substantially altering the percentages of granulocytes and monocytes in the spleen and blood [18]. However, this is not a consistent finding. In the condition of obesity, PLX3397 (50 mg/kg) provided via oral gavage every second day for 21 days significantly decreased the numbers of macrophages in the adipose tissue, but not of circulating myeloid cells [31]. A PLX5562 diet at a concentration of 1200 mg/kg for 21 days significantly depleted macrophages and monocytes in the circulation and liver [25]. One interpretation of these disparate phenomena is that different doses and durations of PLX3397 treatment were used in different studies. It has also been recently reported that PLX3397 treatment at a high dose of 400 mg/kg significantly altered blood cell phenotyping [32]. Specifically, the numbers of red blood cells, hemoglobin, platelets, dendritic cells and Ly6C^−^ monocytes were significantly reduced during microglial depletion via PLX3397 treatment [32]. One recent study suggested that CSF1R inhibitor PLX5622 is not CNS-specific, with peritoneum, lung, and liver tissue macrophages being affected, with CD45^+^CD11b^+^CD106^+^ splenic macrophages not being influenced by the treatment [26]. In contrast, our results determined that the numbers and percentages of splenic red pulp macrophages were significantly reduced after PLX3397 treatment at a dose of 290 mg/kg for 21 days, which represents a widely used dose in research settings. At this time point, reduced numbers of monocytes in the circulation were also recorded. Collectively, systemic administration of CSF1R inhibitors cannot only deplete microglia in the CNS, but also prohibit the committed precursors of peripheral cells. In this case, the ability of circulating monocytes to contribute to the newly repopulated microglial pool after the treatment of CSF1R inhibitors might be reduced.

Overall, our results suggest that there are significant effects on peripheral immune cells following systemic administration of CSF1R inhibitor PLX3397, which has been mostly ignored previously. Furthermore, conditional genetic microglial depletion also exerts effects on peripheral immunity. These may be due to either tamoxifen injections or additional peripheral targets, but also indicate that not only the intended organ should be considered in the use of cell-specific depletion models. Peripheral immune cells also play an important role in the pathogenesis of many diseases and these peripheral effects may lead to potential adverse effects. In addition, the engraftment of peripheral monocytes into the CNS in certain microglial depletion models has added the complexity. Thus, clinical interpretation of microglial depletion studies in disease conditions should be considered with caution. Further monitoring is needed, particularly in ongoing clinical trials [33].

## 4. Materials and Methods

### 4.1. Ethics Statement

All experiments in this study were approved and performed in accordance with the guidelines from the Swedish National Board for Laboratory Animals and the European Community Council Directive (86/609/EEC) under an ethical permit granted by StockholmNord on 11 July 2019.

### 4.2. Animals

*Cx3cr1*^CreER/+^ (Jax) and *Rosa26*^DTA^ (Jax) mice were purchased from the Jackson Laboratory. *Cx3cr1*^CreER/+^ mice and *Rosa26*^DTA^ mice were bred to obtain *Cx3cr1*^CreER/+^*Rosa26*^DTA/+^ mice that were used for conditional genetic microglial depletion. *Cx3cr1*^CreER/+^ mice served as the control group. *C57BL/6NTac* mice (Taconic, Denmark) were bred at Karolinska University Hospital. All experimental mice were maintained under a specific pathogen-free, regulated light/dark schedule and temperature conditions. All experimental mice had free access to standard rodent chow and water. For the PLX3397 dose titration experiments, *C57BL/6NTac* female mice aged between 5- and 7-week-old were used, while both male and female mice were used for all other experiments.

### 4.3. Tamoxifen Treatment

To induce the Cre recombinase in *Cx3cr1*^CreER/+^*Rosa26*^DTA/+^ mice, animals were treated with tamoxifen (TAM; Sigma, T5648-1G, St Louis, USA). Tamoxifen was suspended in the corn oil (Sigma, C8267-500ML, St Louis, MO, USA). Both *Cx3cr1*^CreER/+^*Rosa26*^DTA/+^ and *Cx3cr1*^CreER/+^ mice were administered 5 mg (200 μL) tamoxifen subcutaneously on three consecutive days, as previously described by our research group [9,10].

### 4.4. PLX3397 Treatment

PLX3397 (Pexidartinib, HY-16749, MedChemExpress, USA) was formulated into either 290 mg/kg or 75 mg/kg standard diet (provided by SAFE Nutrition Service, France). PLX3397 diet was administered for 7, 18 or 21 consecutive days depending on research purposes to *C57BL/6NTac* mice. Mice in the control group were fed with a normal diet.

### 4.5. Intracisternal Injection of PLX3397

PLX3397 (Selleck, S7818, USA) were dissolved in 10% DMSO and 90% 100 mg/mL Captisol (Selleck, S4592, USA) at a concentration of 100 μM, and were delivered to the cerebrospinal fluid of mice vial intracisternal injection (10 μL per injection) under isoflurane inhalation (once per day for consecutive two days).

### 4.6. Preparation of Single Cell Suspensions from Blood, Spleen and CNS Tissues

Mice were deeply anesthetized by injecting 100 μg pentobarbital intraperitoneally. Blood was collected from the right ventricle prior to perfusion. Briefly, 200 μL blood were collected into tubes containing ethylenediaminetetraacetic acid (EDTA, E7889, Sigma, Sweden), lysed in ACK buffer (A1049201, Gibco) and then centrifuged. The pellet was resuspended in cold PBS and used for staining. Spleens were dissected and cell suspensions prepared by mechanical dissociation in ice-cold PBS by passing through 40 μm cell strainers (734-0002, VWR; Stockholm, Sweden). Mice were perfused through the left cardiac ventricle using ice-cold PBS. Whole brain and spinal cord were removed and minced with a surgical disposable scalpel (Medicarrier AB, Sweden), followed by enzymatic digestion using Collagenase (11088866001, Roche, Sweden) and DNAse (000000010104159001, Roche, Sweden). Myelin was removed using 38% Percoll (P1644-1L, Sigma, Sweden).

### 4.7. Flow Cytometry

Single cell suspensions were plated in 96-well V-bottom plates and stained at 4 °C for 20 min. Dead cells were removed using Live/DeadTM Fixable Near-IR Dead Cell Stain Kit (Invitrogen, Thermo Fisher Scientific, Stockholm, Sweden) in each panel. The following antibody panels were used: 1. For CNS cells analysis, single cell suspensions were incubated with Percp-Cy5.5-CD11b (clone: M1/70, BioLegend, San Diego, CA, USA), PE/Cy7-CD45 (clone: 30-F11, BioLegend, San Diego, CA, USA), PE-Ly6C (clone: HK1.4, BioLegend, San Diego, CA, USA), V450-Ly6G (clone: 1A8, BD Biosciences, Sweden), APC-F4/80 (clone: BM8, BioLegend, San Diego, CA, USA) and Alexa Fluor700-MHCII (clone: M5/114.15.2, BioLegend, San Diego, CA, USA). 2. For splenic cells analysis, single cell suspensions were incubated with FITC-CD11b (clone: M1/70, BioLegend, San Diego, CA, USA), PE-Ly6C (clone: HK1.4, BioLegend, San Diego, CA, USA), V450-Ly6G (clone: 1A8, BD Biosciences, Sweden), PE/Cy7-F4/80 (clone: BM8, BioLegend, San Diego, CA, USA), V500-CD4 (clone: RM4-5, BioLegend, San Diego, CA, USA) and PCP5.5-NK1.1 (clone: PK136, BioLegend, San Diego, CA, USA). 3. For blood cells analysis, single cell suspensions were incubated with Percp-Cy5.5-CD11b (clone: M1/70, BioLegend, San Diego, CA, USA), PE/Cy7-CD45 (clone: 30-F11, BioLegend, San Diego, CA, USA), PE-Ly6C (clone: HK1.4, BioLegend, San Diego, CA, USA), V450-Ly6G (clone: 1A8, BD Biosciences, Sweden), APC-F4/80 (clone: BM8, BioLegend, San Diego, CA, USA) and FITC-CX3CR1 (clone: SA011F11, BioLegend, San Diego, CA, USA). Cells were acquired using a Gallios flow cytometer (Beckman Coulter, Indianapolis, USA) and analyzed using Kaluza software (Beckman Coulter, Indianapolis, USA).

### 4.8. Statistical Analysis

Statistical analysis was conducted using GraphPad software 8 (San Diego, CA, USA). Comparisons between two groups were made with Mann–Whitney tests. Comparisons of data among multiple groups were analyzed by one-way analysis of variance (ANOVA). Error bars are presented as SEM. Differences at *p* < 0.05 were considered to be statistically significant.

## Supplementary Materials

Supplementary materials can be found at https://www.mdpi.com/1422-0067/21/22/8603/s1. Supplementary Figure S1: Splenic flow cytometry gating strategy. Supplementary Figure S2: Gating strategy of monocytes in the circulation. Supplementary Figure S3: Lower dose of PLX3397 treatment fails to effectively deplete microglia.

## Abbreviations

CNS	Central nervous system
DTR	Diphtheria toxin receptor
DTA	Diphtheria toxin subunit A
