# Supplementary material for: Underestimated Peripheral Effects Following Pharmacological and Conditional Genetic Microglial Depletion
Source: Int J Mol Sci. 2020 Nov 15;21(22):8603. doi: 10.3390/ijms21228603 (PMC7696443; doi:10.3390/ijms21228603)
Supplement: Supplementary file 1 [file ijms-21-08603-s001.pdf]

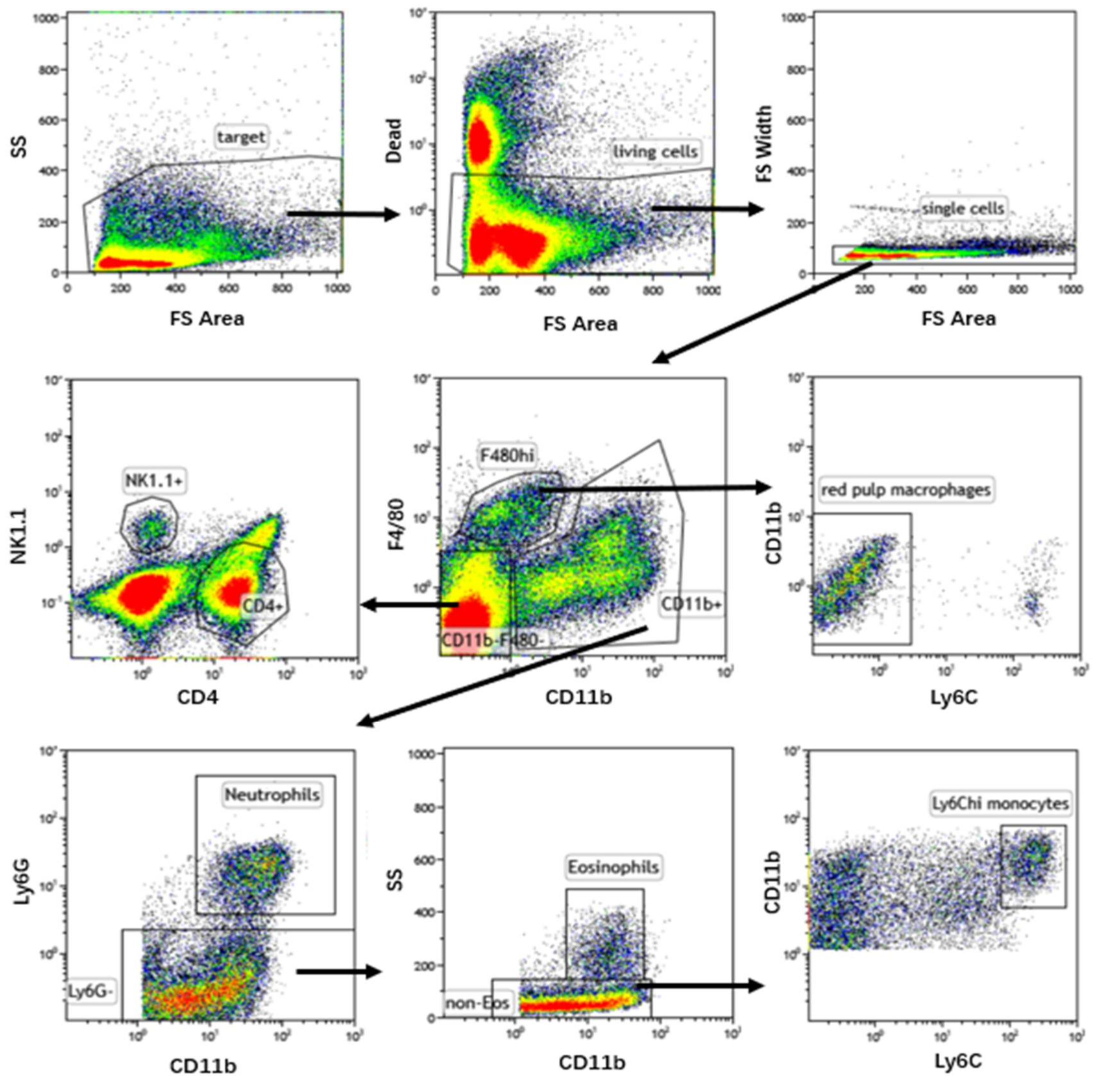

Supplementary Figure S1**:** Splenic flow cytometry gating strategy.

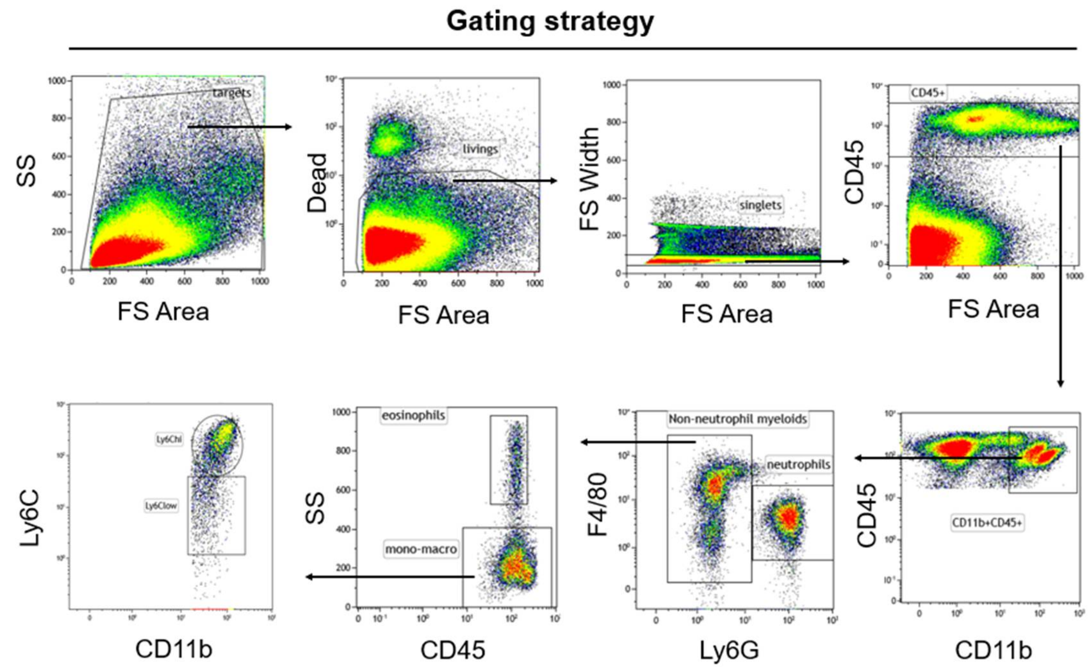

***Supplementary Figure S2*:** Gating strategy of monocytes in the circulation.

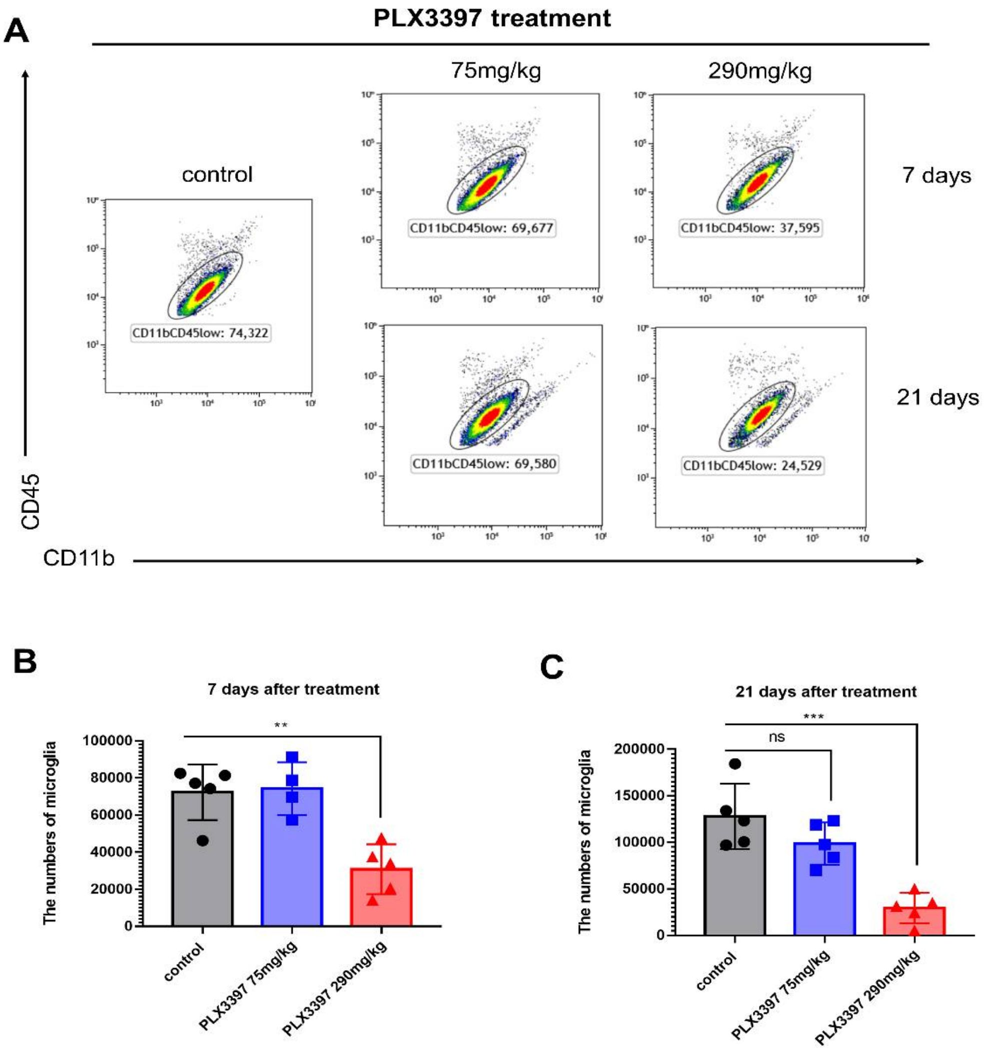

***Supplementary Figure S3*: Lower dose of PLX3397 treatment fails to effectively deplete microglia**. **(A)** Representative flow cytometry plots of CD11b$^+$CD45^{low}Ly6C$^-$Ly6G$^-$ microglia of the hemi-brains in *C57BL/6NTac* mice following consecutive PLX3397 diet (7 and 21 days) with two distinct doses of 75mg/kg and 290mg/kg, respectively. Control mice were treated with normal diet. **(B)** Total CD11b$^+$CD45^{low}Ly6C$^-$Ly6G$^-$ microglial counts (±SEM) of the hemi-brains 7 days following PLX3397 treatment (control, black bars; PLX3397 treatment with the dose of 75mg/kg, blue bars; PLX3397 treatment with the dose of 290mg/kg, red bars). (**C**) Total CD11b$^+$CD45^{low}Ly6C$^-$Ly6G$^-$ microglial counts (±SEM) of the hemi-brains 21 days following PLX3397 diet with a dose of 75mg/kg (control, black bars; PLX3397 treatment with the dose of 75mg/kg, blue bars; PLX3397 treatment with the dose of 290mg/kg, red bars). Statistical significance is indicated as $^*p < 0.05$, $^{**}p < 0.01$ and $^{***}p < 0.001$.
